# Alterations of the Hippocampal Neurogenic Niche in a Mouse Model of Dravet Syndrome

**DOI:** 10.3389/fcell.2020.00654

**Published:** 2020-07-21

**Authors:** Soraya Martín-Suárez, Oihane Abiega, Ana Ricobaraza, Rubén Hernandez-Alcoceba, Juan Manuel Encinas

**Affiliations:** ^1^The Neural Stem Cell and Neurogenesis Laboratory, Achucarro Basque Center for Neuroscience, Leioa, Spain; ^2^Gene Therapy Program CIMA, IdiSNA, Navarra Institute for Health Research, University of Navarra, Pamplona, Spain; ^3^Department of Neurosciences, Faculty of Medicine and Nursing, University of the Basque Country (UPV/EHU), Leioa, Spain; ^4^IKERBASQUE, The Basque Foundation for Science, Bilbao, Spain

**Keywords:** neural stem cells, aberrant neurogenesis, gliosis, Dravet syndrome, SCN1A

## Abstract

Hippocampal neurogenesis, the process by which neural stem cells (NSCs) continuously generate new neurons in the dentate gyrus (DG) of most mammals including humans, is chiefly regulated by neuronal activity. Thus, severe alterations have been found in samples from epilepsy patients and in the hippocampal neurogenic niche in mouse models of epilepsy. Reactive-like and gliogenic NSCs plus aberrant newborn neurons with altered migration, morphology, and functional properties are induced by seizures in experimental models of temporal lobe epilepsy. Hippocampal neurogenesis participates in memory and learning and in the control of anxiety and stress. It has been therefore hypothesized that part of the cognitive symptoms associated with epilepsy could be promoted by impaired hippocampal neurogenesis. We here analyze for the first time the alterations of the neurogenic niche in a novel mouse model of Dravet syndrome (DS), a genetic encephalopathy with severe epilepsy in infancy and multiple neurological comorbidities. Scn1a^WT/A1783V^ mice, hereafter referred to as DS, carrying a heterozygous and clinically relevant SCN1A mutation (A1783V) recapitulate the disease at the genetic and phenotypic levels. We demonstrate that in the neurogenic niche of young adult DS mice there are fewer NSCs, they have impaired cell division and bear reactive-like morphology. In addition, there is significant aberrant neurogenesis. Newborn immature neurons migrate abnormally, and several morphological features are drastically changed. Thus, this study shows for the first time important modifications in hippocampal neurogenesis in DS and opens venues for further research on this topic.

## Introduction

The hippocampus is one of the most vulnerable structures of the brain to excitotoxicity and particularly to seizures due to its recurrent circuits. The hippocampus of most mammals, including humans (Eriksson et al., 1998; Moreno-Jiménez et al., 2019), is able to generate new neurons through adulthood and aging through a complex process termed adult hippocampal neurogenesis (AHN). However, excitotoxicity and seizures impair AHN with several consequences: (a) the neurons lost by excitotoxicity cannot be regenerated; (b) the normal functions of AHN are disrupted; and (c) the normally neurogenic neural stem cells (NSCs) of the hippocampus turn to participate in reactive gliogenesis. The persistence of AHN in the dentate gyrus (DG) of the hippocampus is consequence of a population of NSCs with neurogenic (Seri et al., 2001) and gliogenic capacity (Encinas et al., 2011). NSCs remain quiescent until they get activated to enter the cell cycle and then generate neuronal precursors during a short period of time. After several weeks of migration and differentiation, newborn neurons get finally integrated into the hippocampal circuitry modifying the properties of the pre-existent neuronal network (van Praag et al., 2002; Aimone et al., 2014). Newborn neurons participate in the formation of new memories (Farioli-Vecchioli et al., 2008); in learning (Zhang et al., 2008; Deng et al., 2009); in the responses to stress, anxiety, and fear (Santarelli et al., 2003; Saxe et al., 2006; Bergami et al., 2008; Zhang et al., 2008; Aimone et al., 2011; Snyder et al., 2011), as well as in spatial and object recognition memory (Jessberger et al., 2009) and in pattern separation (Sahay et al., 2011; Nakashiba et al., 2012). Shortly after being activated to generate neuronal precursors, NSCs undergo ultimate gliogenic (Bonaguidi et al., 2011; Encinas et al., 2011) or neurogenic (Pilz et al., 2018) differentiation. Thus, the population of NSCs gets depleted overtime in an activation-dependent manner so that the population, and therefore AHN, is maximal during the postnatal-juvenile period, and is minimal in the aged brain (Sorrells et al., 2018).

Previous studies based on epilepsy models in adult mice have shown that AHN can be affected in two manners dependent on the level of neuronal hyperactivation (Bielefeld et al., 2019): With lower levels of epileptic activity and epileptiform activity, newborn neurons are the cell type that results more affected. They migrate abnormally, they extend abnormal basal dendrites, and their physiological properties are modified (Parent et al., 1997). Together, this set of alterations has been referred to as aberrant neurogenesis. On the other hand when using models of mesial temporal lobe epilepsy (MTLE), in which kainic acid is delivered into the hippocampus (Bouilleret et al., 1999; Sierra et al., 2015) or the amygdala (Muro-García et al., 2019) and the epileptic focus is unilaterally localized in this structure, a more dramatic alteration is induced. NSCs abandon their neurogenic program and transform into reactive-NSCs (React-NSCs) which ultimately differentiate into reactive astrocytes (RAs) that contribute to hippocampal reactive gliosis (Sierra et al., 2015; Muro-García et al., 2019). As a result, a small amount of aberrant neurogenesis or no neurogenesis at all is found (Murphy et al., 2012; Sierra et al., 2015; Muro-García et al., 2019).

We hypothesize that because the population of NSCs and AHN are maximal in postnatal and juvenile stages, the effects of seizures in infant epilepsy on the neurogenic niche could have a greater impact on these earlier stages than in later ones. Dravet syndrome (DS, OMIM 607208), first described in 1978 by Charlotte Dravet (Dravet, 1978, 2011) and also known as severe myoclonic epilepsy of infancy (SMEI), is a rare but severe form of epilepsy affecting approximately one in 15,000 births (Dravet et al., 2005; Wu et al., 2015). DS characterizes by the early onset (4–6 months of age) of recurrent generalized seizures in previously healthy children, followed by frequent prolonged seizures that are resistant to anti-epileptic drugs. As a result, the symptoms include developmental delays, speech impairment, dysautonomia, nutrition issues, autism-like behavior, and a high rate of sudden unexpected death in epilepsy (SUDEP). Approximately 10–20% of the afflicted patients die prematurely (Hurst, 1990; Dravet et al., 2005; Jansen et al., 2006; Genton et al., 2011; Sakauchi et al., 2011). Almost the 90% of cases with DS present *de novo* mutations in the SCN1A gene, encoding the alpha subunit of a voltage-gated sodium channel type 1 (Nav1.1) (Oguni et al., 2005; Korff and Nordli, 2006; Catterall et al., 2008; Depienne et al., 2009; Marini et al., 2009; Mullen and Scheffer, 2009). Newer inducible mouse models have been generated that overcome the drawbacks of the previous constitutive models which were biased toward the subset of DS mice that survived and could breed (Williams et al., 2019). We here analyze for the first time the alterations of the hippocampal neurogenic niche in the previously characterized C57BL/6J knock-in mouse strain (Scn1a^WT/A1783V^, DS). These mice carry a clinically relevant heterozygous SCN1A mutation (A1783V) and present the full spectrum of DS manifestations (Ricobaraza et al., 2019). Increased susceptibility to hyperthermia-induced seizures (applied to minimize variability in the data derived from spontaneous seizures) starts at postnatal week 3, before the period of maximal mortality rate at 8 weeks of life (Ricobaraza et al., 2019). One of the mechanisms suggested to trigger the onset of seizures at that age is the increase of Nav1.1 expression compensating the normal decline of Nav1.3 early after birth as observed in humans (Cheah et al., 2013). Although the physiopathology of DS is complex and not fully elucidated, the most accepted mechanism is the impaired excitability of parvalbumin and somatostatin-expressing GABAergic interneurons (Tai et al., 2014) which tips the neurotransmission balance in favor of excitation.

## Materials and Methods

For an extended description of methods, see sections “Materials and Methods” and “Supplementary Materials and Methods” in Supplementary Material.

### Animals

The conditional *Scn1a*-A1783V mice [B6(Cg)-Scn1atm1.1Dsf/J, The Jackson Laboratory, stock no. 026133] were bred to mice expressing Cre recombinase under the control of the CMV promoter [B6.CTg(CMV-Cre)1Cgn/J, The Jackson Laboratory, stock no. 006054]. Breeding pairs consisted of heterozygous male *Scn1a*-A1783V and homozygous female CMV-Cre mice. See https://www.jax.org/strain/026133 for details about allele modification and genotyping. Offspring carrying one mutated allele (genotype hereinafter referred to as Scn1a^WT/A1783V^ and the mice referred to as DS mice throughout the text) express the A1783V mutation in the *Scn1a* gene in all body tissues, mimicking what happens in DS. Animals were housed four to six per cage with free access to food and water, weighed weekly, and maintained in a temperature and light controlled (12/12 h light/dark cycle) environment. The studies were performed by comparing heterozygous transgenic Scn1a^WT/A1783V^ to age-matched negative littermates Scn1a^WT/WT^ (referred to as WT mice throughout the text). Breeding and experimental protocols were approved by the Ethical Committee of the University of Navarra (in accord with the Spanish Royal Decree 53/2013). These animals have been previously characterized (Ricobaraza et al., 2019). Five age-matched (7 weeks) animals were used for each group.

### Immunohistochemistry

Immunohistochemical, image capture, and quantification techniques were performed essentially as described before following methods optimized for the use in transgenic mice (Encinas and Enikolopov, 2008; Encinas et al., 2011; Sierra et al., 2015; Muro-García et al., 2019).

### Statistical Analysis

SigmaPlot (San Jose, CA, United States) was used for statistical analysis. A Student’s *t*-test was performed in all cases to compare data from WT to DS mice for all quantifications except for data from the 3D-Sholl analysis in which a repeated-measures two-way ANOVA with a Bonferroni *post hoc* test was employed. When variances were not homogeneous (by Levene’s test) a Mann–Whitney rank sum test was used instead. This case only happened for the data of neuroblasts with basal dendrites. Only *p* < 0.05 is reported to be significant. Data are shown as mean ± standard error of the mean (SEM).

## Results

**FIGURE 1:**
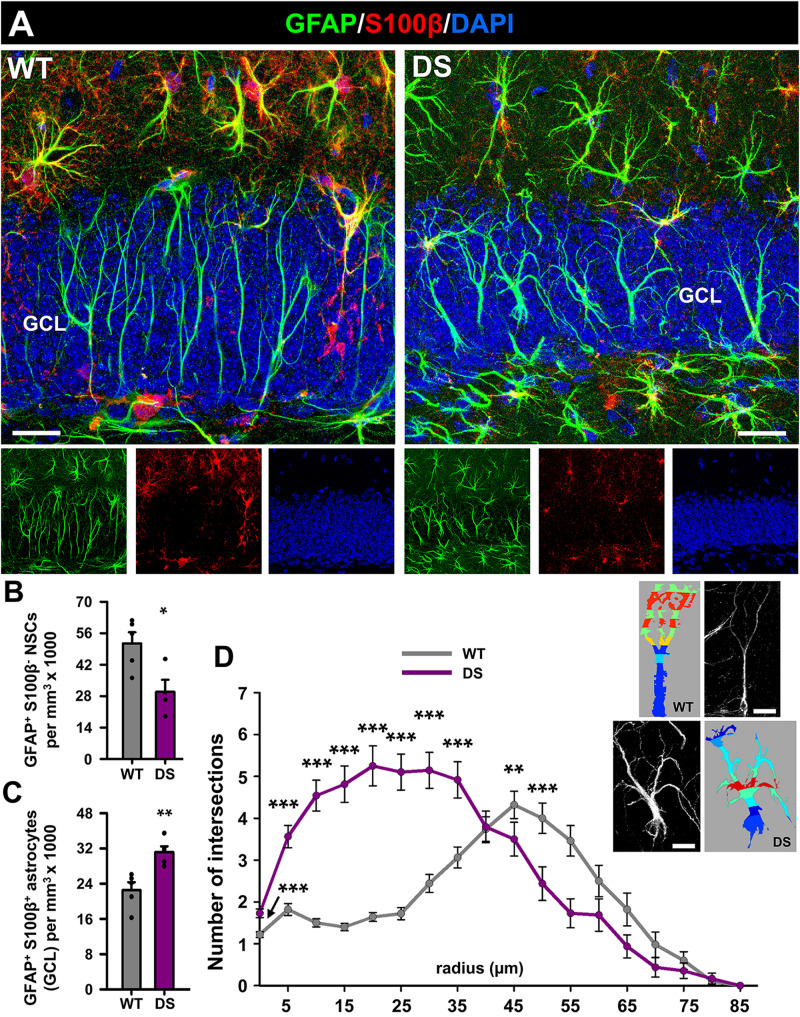
Reactive morphology of NSCs in DS mice. **(A)** Representative confocal-microscopy images of the DG of WT (left) and DS (right) mice after staining for GFAP (NSCs and astrocytes) and S100β (astrocytes). DAPI was used to stain cell nuclei. The number of NSCs is significantly decreased in DS mice **(B)** but the number of astrocytes is higher **(C)**. *p < 0.05, **p < 0.01, by Student’s *t*-test **(B,C)**. The morphology of NSCs was studied by 3D-Sholl analysis using reconstructed cells derived from confocal microscopy z-stacks (insets) and showed an increase in the number of intersections in DS mice **(D)** **p < 0.01, ***p < 0.001 by repeated-measures two-way ANOVA followed by Bonferroni *post hoc* test in. Bars show mean ± SEM. Dots show individual data. *n* = 5 for each condition. Scale bar in **(A)** is 20 μm and 10 μm in **(B)**.

We first sought to characterize the properties of NSCs in WT and DS mice. For that purpose, we co-stained brain slices from 7-week-old WT or DS mice with antibodies against GFAP, which is expressed by both astrocytes and NSCs, and against S100β, which is present in astrocytes and RAs but not in NSCs (Figure 1A). We centered the analysis in the granule cell layer and the subgranular zone (GCL+SGZ) to further assure the identity of NSCs. We first quantified the number of NSCs (GFAP^+^ S100β^–^-cells in the GCL+SGZ) and found a statistically significant decreased number in the DS compared to the WT mice (Figure 1B). In contrast, the number of astrocytes (GFAP^+^ S100β^+^-cells) in the area of interest was significantly increased (Figure 1C). We further analyzed the morphology of NSCs by 3D-Sholl analysis as increased morphological complexity is a hallmark of reactive NSCs (React-NSCs). Indeed, NSCs in the DS mice had clearly altered morphology with significantly higher complexity (Figure 1D and Supplementary Figure S1A). These results suggest that similarly to what is found in adult mouse models of MTLE, NSCs also develop a reactive morphology in DS. As we observed an increased number of astrocytes and increased overall intensity of staining for GFAP and S100β (Supplementary Figure S1A) we further analyzed the presence of gliosis. The expression of GFAP was significantly increased in the SGZ+GCL as measured by the area occupied by GFAP^+^-pixels (Figure 1B). We therefore used S100β as a specific astrocytic marker, because GFAP is also expressed in NSCs, and measured the area occupied by S100β^+^-pixels in the GCL+SGZ. We found that it was significantly increased in the DS mice compared to the WT ones (Supplementary Figure S1B). The same result was found when we measured the integrated density of the S100β^+^-pixels (Supplementary Figure S1C). The morphological complexity of S100β^+^-cells measured by 3D-Sholl analysis was also higher in the DS animals versus the WT (Supplementary Figure S1D). We also observed a slight increase in the span of the GCL, using DAPI nuclear staining (Supplementary Figure S1E), and measuring the distance occupied by nuclei from the hilus to the molecular layer (Supplementary Figure S1F). GCL dispersion normally accompanies gliosis in human and experimental models of MTLE.

**FIGURE 2:**
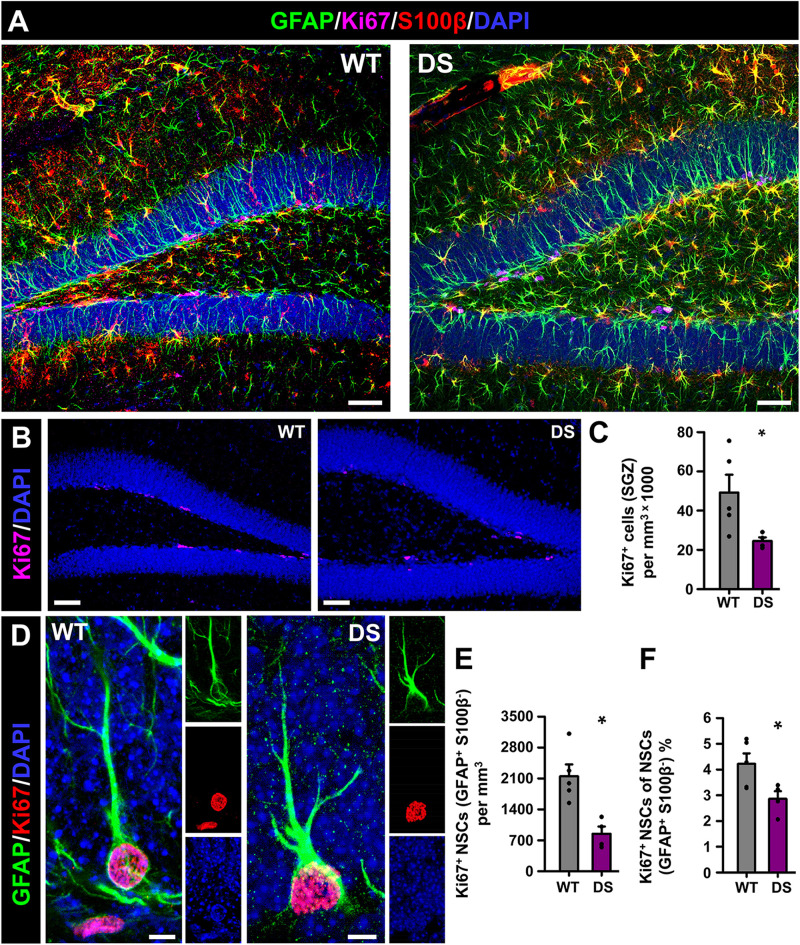
Alterations NSC activation in DS mice. **(A)** Representative confocal-microscopy images of the DG of WT (left) and DS (right) mice after staining for GFAP (NSCs and astrocytes), S100β (astrocytes), and the proliferation marker Ki67. DAPI was used to stain cell nuclei. Ki67^+^-cells were restricted to the SGZ in WT and DS mice **(B)**. Fewer proliferating cells (Ki67^+^) were found in the DS mice **(C)**. **(D)** High-magnification confocal-microscopy images of dividing NSCs in WT (left) and DS (right, note the ramified morphology). The total number **(E)** as well as the proportion of dividing (Ki67^+^) NSCs was lower in the DS mice **(F)**. *p < 0.05 by Student’s *t*-test. *n* = 5 for each condition. Bars show mean ± SEM. Dots show individual data. Scale bar in **(A)** is 40 μm, 50 μm in **(B)** and 5 μm in **(D)**.

We moved next to investigate cell proliferation in the neurogenic niche and the cell division of NSCs in particular. In this case, we co-stained brain slices from 7-week-old WT and DS mice for GFAP, S100β^+^, and for Ki67, a marker of cells undergoing mitosis (Figure 2A). The total number of dividing cells (Ki67^+^-cells), which were only found in the SGZ (Figure 2B), was significantly lower in the DS mice than in the WT mice (Figure 2C). The number of dividing NSCs was also diminished in the DS mice (Figures 2D,E), with a significant drop in the proportion of the already normally low subset of dividing NSCs (Figure 2F). Together these results show that hippocampal NSCs show reactive morphology plus impaired activation to enter the cell cycle in DS mice.

**FIGURE 3:**
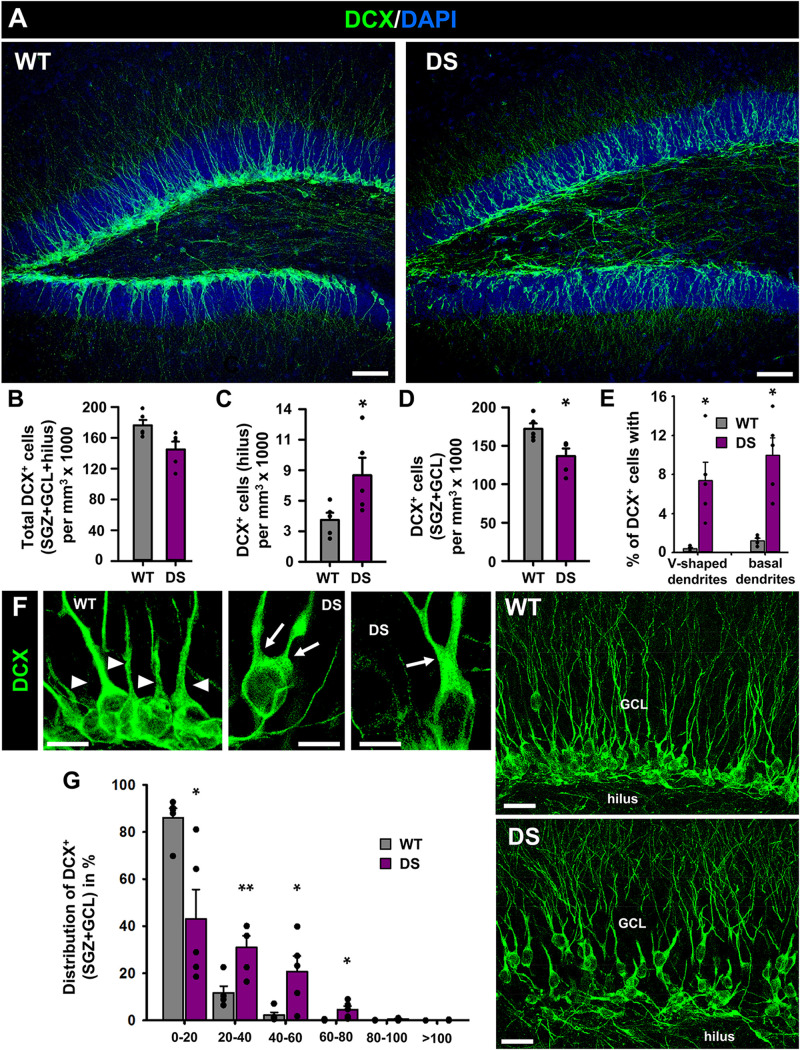
Aberrant neurogenesis in DS mice. **(A)** Confocal-microscopy images of the DG of WT (left) and DS (right) immunostained for DCX (a specific marker of neuroblasts) and stained for DAPI. There are no differences in the overall number of DCX^+^-cells **(B)** but there are more neuroblasts in the hilus of DS mice **(C)** and correspondingly fewer in the SGZ+GCL **(D)**. In addition, neuroblasts in the DS mice presented with much higher frequency a V-shaped apical dendrite (two dendrites emerging directly from the soma or the main one bifurcating within the first 10 μm) (**E**, left) and basal dendrites (**E**, right), as can be visualized in higher magnification images **(F)**. The arrowheads point toward single dendrites emerging from individual neuroblasts whereas arrows point toward two dendrites emerging directly from the soma or bifurcating in the most proximal segment. The images in the right show the abnormal migration of neuroblasts. In the DS mice, DCX^+^-cells had migrated to upper layers of the GCL, closer to the molecular layer whereas in the WT mice they are localized in the SGZ **(G)**. *p < 0.05, **p < 0.01, by Student’s *t*-test. *n* = 5 for each condition. Bars show mean ± SEM. Dots show individual data. Scale bar in **(A)** is 40 μm and 10 μm in **(F)** (left) and 20 μm in **(E)** (right).

To test whether these alterations were also accompanied by changes in neurogenesis we stained brain slices from 7-week-old WT and DS mice for doublecortin (DCX) (Figures 3A,E), a specific marker of neuroblasts (immature neurons). We found different effects: The overall number of DCX^+^ neuroblasts was not significantly changed, although a trend toward a lower number was found in the DS mice versus the WT (Figure 3B); numerous neuroblasts were, however, found in the hilus of DS mice, which is very unusual in normal conditions (Figure 3C). In contrast, significantly fewer neuroblasts were located to the GCL+SGZ (Figure 3D). Further analysis at higher magnification (Figure 3E) allowed the observation of morphological alterations in neuroblasts of the DS mice. An increased proportion of DCX^+^ neuroblasts presented a V-shaped apical dendrite (defined as two dendrites emerging directly from the soma or the main one bifurcating within the first 10 μm) instead of the normal single main dendrite emerging from the soma (Figures 3E,F-left). In addition, a high proportion of them showed basal dendrites, which neuroblasts rarely extend in normal conditions (Figures 3E,F-right). Also, DCX^+^ neuroblasts showed abnormal migration in the DS mice, being distributed in the higher layers of the GCL, toward the molecular layer and away from the SGZ where they are normally positioned (Figures 3E,G). Increased quantity of immature neurons with abnormal migration and abnormal morphology is one of the hallmarks of aberrant neurogenesis described in adult models of MTLE (Parent et al., 1997). No DCX^+^ neuroblasts were observed in other areas such as the enthorinal cortex or the amygdala in which cellular plasticity in the form of neurogenesis has been proposed to take place.

**FIGURE 4:**
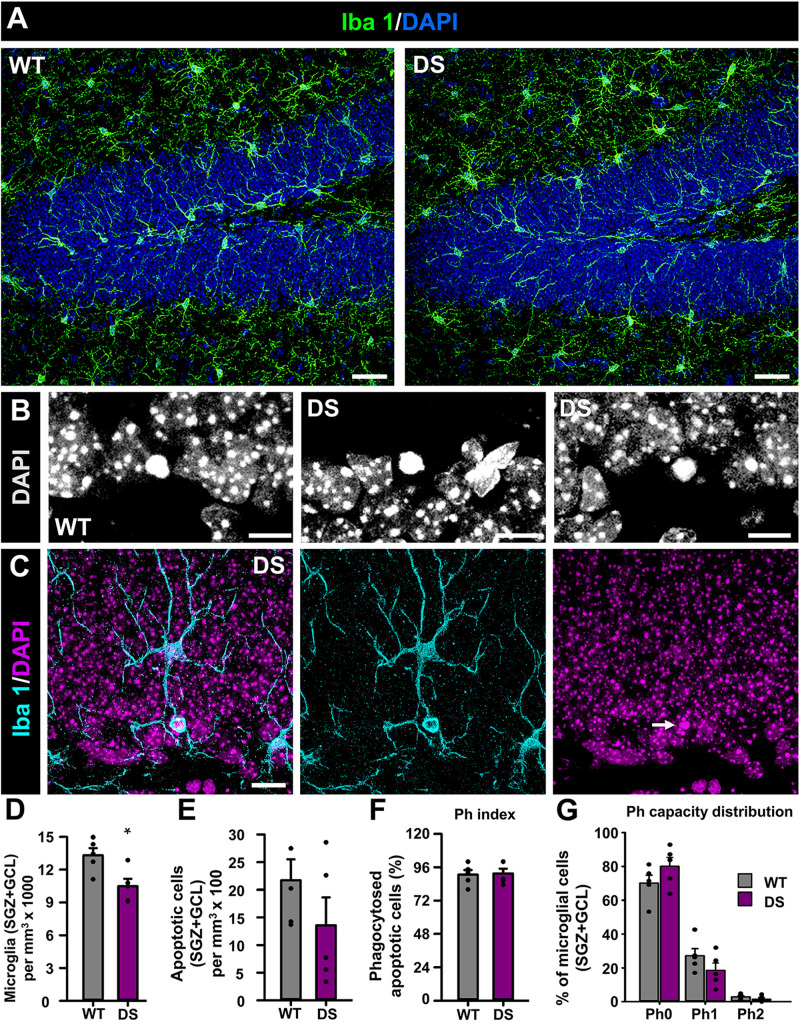
Alteration of microglia in DS mice. **(A)** Confocal-microscopy images of the DG of WT (left) and DS (right) immunostained for Iba 1 (a specific marker of macroglia in the brain) and stained for DAPI. DAPI DNA staining was used to identify apoptotic (condensed) nuclei at higher magnification **(B)**. Phagocytosis was identified as microglial pouches extended form microglia cells (Iba 1^+^) wholly engulfing apoptotic nuclei **(C)**. There were fewer microglia in the SGZ+GCL of DS mice **(D)**. The number of apoptotic nuclei did not change from WT to DS mice **(E)**. The number of apoptotic cells being engulfed by microglia was not altered either **(F)**. There were not changes in the proportion of microglia with either none, 1, or 2 phagocytosing poaches (Ph) **(G)**. *p < 0.05 by Student’s *t*-test in **(D)**. *n* = 5 for each condition. Bars show mean ± SEM. Dots show individual data. Scale bar in **(A)** is 40 μm, 10 μm in **(B)**, and 20 μm in **(C)**.

Finally, we wanted to test whether microglia could be also altered in the DG of DS mice as modifications have been reported in human and mouse models of MTLE (Abiega et al., 2016). We stained brain slices from 7-week-old WT and DS mice with the specific microglial marker Iba 1 (Figure 4A) and used the nuclear staining DAPI to identify apoptotic cells (Figures 4B,C) (Sierra et al., 2010). We first found that the number of microglia, measured in the GCL+SGZ was slightly but significantly reduced in DS mice compared to WT (Figure 4D). Next, we sought to test potential alterations in the functional properties of microglia in the neurogenic niche (Sierra et al., 2010; Abiega et al., 2016) using phagocytosis as a readout (Figure 4C). We first checked the number of apoptotic cells in the GCL+SGZ (Figure 4E) as well as the phagocytic index (percentage of apoptotic cells that are being phagocytosed by microglia) (Figure 4F) and then the phagocytosis capacity distribution [percentage of microglia with 0, 1, or 2 phagocytosing poaches (Ph)] (Figure 4G). Interestingly, none of these parameters were different in the DS mice when compared to the WT animals.

## Discussion

We herein describe for the first time the alterations of the young adult hippocampal neurogenic niche in a recently characterized mouse model of DS, the Scn1a^WT/A1783V^ mice (Ricobaraza et al., 2019). This transgenic line is a conditional C57BL/6 J knock-in mouse that carries the heterozygous and clinically relevant SCN1A mutation (A1783V) and presents a full spectrum of DS manifestations. These include cognitive/behavioral impairment; spontaneous interictal epileptiform activity; and reduced threshold for heat-induced seizures. Seizures are multifocal with intra as well as interhemispheric propagation and generate generalized tonic–clonic seizures. In addition, the Scn1a^WT/A1783V^ mice have a 70% mortality rate within the first 8 weeks of age and this is the reason why we chose the 7-week time point as the center of our study.

AHN has been shown to participate in cognitive tasks related to spatial memory and associative learning (Saxe et al., 2006; Dupret et al., 2008; Farioli-Vecchioli et al., 2008; Imayoshi et al., 2008; Clelland et al., 2009; Deng et al., 2009), as well as in the responses to stress and depression (Snyder et al., 2011). One of the main regulators of AHN is neuronal activity and subsequently is greatly affected by seizures. Thus, it has been hypothesized that part of the cognitive symptoms found in epilepsy patients, such as memory impairment (Gargaro et al., 2013), anxiety, and depression (Heuser et al., 2009) could be provoked or enhanced by defects in AHN. We further argue that the negative effect of seizures on AHN can be greater if epileptic seizures start when hippocampal neurogenesis is maximal, i.e., in early postnatal periods, as it is the case of DS. Earlier works focused mostly on the alterations caused by seizures on differentiating newborn neurons. Amygdala kindling and pilocarpine-induced epileptic seizures induced “aberrant neurogenesis”: neurons were generated in excess, they migrated abnormally (moving into the hilus and molecular layer instead of the GCL) and presented morphological anomalies such as basal dendrites (Parent et al., 1997, 1998). These pioneering studies also found ectopic GC neurons in the hilus and molecular layer in the DG of epilepsy patients (Parent et al., 2006). Other study, using KA as inductor of seizures, suggested that NSCs could also be affected by seizures, as their rate of cell division was increased, reporting also that gliogenesis was also augmented (Huttmann et al., 2003).

We have recently shown, using an intrahippocampal and an intramygdalar injection of KA as models of MTLE, that NSCs are dramatically affected by seizures and that the neurogenic niche is severely disrupted too (Sierra et al., 2015; Muro-García et al., 2019). We have previously discussed that the different levels of neuronal hyperexcitation will affect the neurogenic niche differentially (Bielefeld et al., 2019): Milder events would translate into a temporary increase of progenitor proliferation without other changes but more extreme ones, such as the case of experimental MTLE, can induce NSCs to contribute to reactive gliosis by converting into RAs at the expense of abandoning their neurogenic program. In the DS mice, we found that NSCs presented reactive morphology and that there was increased astrogliosis. In contrast with MTLE, NSCs divided with lower frequency. This finding could be attributed as them being in the second phase of reduced activation, due to astroglial differentiation and neuroinflammatory factors, after the early massive activation triggered by the initial episodes of seizures (Huttmann et al., 2003; Sierra et al., 2015; Muro-García et al., 2019). Future extensive time-course experiments and lineage-tracing analysis would help clarify whether indeed an initial phase of NSC overactivation and increased astroglial differentiation takes place. Long-term studies will show whether neurogenesis drops below basal levels and whether it remains aberrant.

At any rate, the level of reactive gliosis in the DS model is lower than that found in MTLE, and it is actually permissive of AHN as shown by the presence of DCX-expressing immature neurons (neuroblasts). The overall number of neuroblasts was not actually changed in comparison to controls, but typical features of aberrant neurogenesis (Parent et al., 1997, 1998; Jessberger et al., 2007) were found. We speculate that due to the conversion of NSCs into reactive-like NSCs with reduced rate of cell division, neurogenesis would be depleted in the long term if the animals survived more than 8 weeks. Regardless of the total numbers of neuroblasts, it is clear that their morphology and location are abnormal. These changes have been shown to correlate with changes in their synaptic functional properties (Pierce et al., 2011) and represent a major finding in this study. The features of aberrant neurogenesis described herein are similar to those induced by stress and knockdown of glucocorticoid receptors (Fitzsimons et al., 2013). At this stage, however, and due to lack of studies on this issue, we cannot make any claim about this mechanism driving aberrant neurogenesis in this DS model. In spite of the controversy regarding the existence of neurogenesis in adult and aged humans with strong arguments in favor and against—see Lucassen et al. (2019) and Petrik and Encinas (2019) for critical reviews—the data available coincide in that neurogenesis is maximal during the early postnatal periods, first weeks in mice, and first years in humans, exactly the period in which seizures start and are more frequent in DS.

Seizures in MTLE models also increase apoptosis in the neurogenic niche and neighboring regions (Bengzon et al., 1997; Muro-García et al., 2019), and microglia has been shown to become severely impaired (Abiega et al., 2016). In the Scn1a^A1783V^ mice, we have only encountered a slight decrease in the number of microglia in the neurogenic niche, but none of the functional parameters related to phagocytosis were altered. These results are somehow expected, as neuronal loss is not a prominent characteristic in DS (Le Gal et al., 2010; Hata et al., 2020), which in turn can be due to the absence of massive reactive gliosis and neuroinflammation in the DS hippocampus. We hypothesize that this is a reflection of the lower level of neuronal excitotoxicity and neuronal death localized in the DG due to the variable origin of seizures in comparison with the maximal level of hyperexcitation and excitotoxicity triggered by on-site hippocampal seizures typical of MTLE. Indeed, cell death was not changed in DS mice, providing a first explanation as to why microglia is mostly unchanged. In this scenario, astrocytes and NSCs would be more sensitive to changes in neuronal activity than microglia, an aspect that remains to be explored. The study of the previous mouse models of DS was mainly focused in the alterations of neuronal electrophysiological properties and the characterization of the newer inducible models of DS have been focused on also neuronal activity and behavior tasks (Ricobaraza et al., 2019; Williams et al., 2019). Thus, the mechanisms for reactive gliosis and for the induction of reactive NSCs and aberrant neurogenesis remain unexplored as they have only been recently described.

## Data Availability Statement

The raw data supporting the conclusions of this article will be made available by the authors, without undue reservation.

## Ethics Statement

The animal study was reviewed and approved by Ethical Committee of the University of Navarra.

## Author Contributions

SM-S participated in the experimental design, performed the experiments and data analysis, prepared the figures, and co-wrote the manuscript. OA participated in the experimental design, performed the experiments and data analysis, and prepared the figures. AR participated in the experimental design, generated the mice, provided the samples, and helped writing the manuscript. RH-A supervised sample collection and helped writing the manuscript. JE designed the project, participated in the experimental design, performed the experiments and data analysis, prepared the figures, provided funding, and wrote the manuscript. All authors contributed to the article and approved the submitted version.

## Conflict of Interest

The authors declare that the research was conducted in the absence of any commercial or financial relationships that could be construed as a potential conflict of interest.
