# Supplementary material for: Alterations of the Hippocampal Neurogenic Niche in a Mouse Model of Dravet Syndrome
Source: Front Cell Dev Biol. 2020 Jul 21;8:654. doi: 10.3389/fcell.2020.00654 (PMC7385077; doi:10.3389/fcell.2020.00654)
Supplement: Supplementary file 1 [file Data_Sheet_1.pdf]

Alterations of the hippocampal neurogenic niche in a mouse model of Dravet syndrome

Supplementary Materials and Methods

Animals

The conditional *Scn1a*-A1783V mice (B6(Cg)-Scn1atm1.1Dsf/J, The Jackson Laboratory, stock no. 026133) were bred to mice expressing Cre recombinase under the control of the CMV promoter (B6.CTg(CMV-Cre)1Cgn/J, The Jackson Laboratory, stock no. 006054). Breeding pairs consisted of heterozygous male *Scn1a*-A1783V and homozygous female CMV-Cre mice. See https://www.jax.org/strain/026133 for details about allele modification and genotyping. Offspring carrying one mutated allele (genotype hereinafter referred to as $Scn1a^{WT/A1783V}$ and referred to as DS mice throughout the text) express the A1783V mutation in the *Scn1a* gene in all body tissues, mimicking what happens in DS. Animals were housed 4–6 per cage with free access to food and water, weighed weekly, and maintained in a temperature and light controlled (12 h/12 h light/dark cycle) environment. The studies were performed by comparing heterozygous transgenic $Scn1a^{WT/A1783V}$ to age-matched negative littermates $Scn1a^{WT/WT}$ (referred to as WT mice throughout the text). Breeding and experimental protocols were approved by the Ethical Committee of the University of Navarra (in accord with the Spanish Royal Decree 53/2013). These animals have been previously characterized (Ricobaraza et al., 2019). Five age-matched (7 weeks) animals were used for each group.

Immunohistochemistry

Immunohistochemical techniques were performed essentially as described before following methods optimized for the use in transgenic mice (Encinas and Enikolopov, 2008; Encinas et al., 2011). Animals were subjected to transcardial perfusion with 20 ml of PB followed by 36 ml of 4% (w/v) paraformaldehyde (PFA) in PB, pH 7.4. The brains were removed and postfixed, with the same fixative, for 3 h at room temperature, then transferred to PB and kept at 4ºC. The same fixative was used for overnight postfixation.

Serial 50 µm-thick sagittal sections were cut using a Leica VT 1200S vibratome (Leica Microsystems GmbH, Wetzlar, Germany). Immunostaining was carried out following a standard procedure: the sections were incubated with blocking and permeabilization solution containing 0.25% Triton-X100 and 3% bovine serum albumin (BSA) in PBS for 3 h at room temperature, and then incubated overnight with the primary antibodies (diluted in the same solution) at 4ºC. After thorough washing with PBS, the sections were incubated with fluorochrome-conjugated secondary antibodies diluted in the permeabilization and blocking solution for 3 h at room temperature. After washing with PBS, the sections were mounted on slides with Dako fluorescent mounting medium (Agilent-Dako S3023).

The following primary antibodies were used: goat anti-GFAP (Abcam Ab53554, 1:1,000); mouse anti-S100β (Sigma-Aldrich S2532, 1:1000); goat anti-DCX (Santa Cruz sc-ab53554, 1:500); rabbit anti-Iba 1 (Wako 19-19741, 1:1000); rabbit anti Ki67 (Vector Laboratories VP-RM04, 1:1000). The secondary antibodies used, all from ThermoFisher Scientific and in 1:500 concentration, were: donkey anti-rabbit Alexa Fluor 568 (A10043); donkey anti-mouse Alexa Fluor 647 (A-31571); goat anti-rabbit Alexa Fluor 488 (A-11034) and donkey anti-goat Alexa Fluor 488 (A-21084). 4′,6-diamidino-2-phenylindole (DAPI, Sigma-Aldrich D9542), at 1:1000, was also added to the sections during the incubation with the secondary antibodies to counterstain cell nuclei.

Image capture

All fluorescence immunostaining images were collected employing a Leica SP8 (Leica, Wetzlar, Germany) laser scanning microscope and LAS X software. Images were exported as tiffs and adjusted for brightness, contrast, and background, equally for the entire image, using the "levels" tools in Adobe Photoshop without any further modification. All images shown are flat projections, from z-stacks of approximately 10 μm of thickness.

Cell quantification

Quantitative analysis of cell populations in histological samples was performed by design-based (assumption free, unbiased) stereology using a modified optical fractionator-sampling scheme as previously described (Encinas and Enikolopov, 2008; Encinas et al., 2011). Hemispheres were sliced sagittally in a lateral-to-medial direction including the entire dentate gyrus (DG). Slices were collected using systematic-random sampling. The 50-μm slices were collected in 5 parallel sets, each set consisting of approximately 12 slices, each slice 250 μm apart from the next.

Cells were categorized following the criteria described previously (Encinas and Enikolopov, 2008; Encinas et al., 2011). NSCs were defined as radial glia-like $GFAP^{+}S100\beta^{-}$. Quantification was restricted to the SGZ and the GCL and occasionally (neuroblasts) to the hilus. Neuroblasts were identified by expression of the specific marker doublecortin (DCX). For proportional quantifications we used confocal z-stacks from 30 μm below to 30 μm above the GCL using a 63x oil immersion objective. At least 100-300 cells per animal were identified (NSCs, neuroblasts) and then assigning each one to the subpopulation of interest (proliferating or not NSCs; neuroblast with or without V-shaped apical dendrite or with or without basal dendrites etc.). V-shaped dendrites were scored when two dendrites emerged directly from the soma or the main single one bifurcated withing the first 10 μm proximal to the soma). All absolute quantifications were performed with the 40x magnification, capturing frames of known volume (typically 100w x 100h x 10d). Density was then normalized for the total surface of the SGZ of the DG, measured at 40x. The volume of the DG was not used (the height of the SGZ plus the GCL was excluded) because the GCL might change in DS mice due to cell dispersion.

Astrogliosis.

The area occupied by astrocytes was measured in the SGZ + GCL using the open-source FIJI (Image J). First, $S100\beta^{+}$-cells were manually selected in z-stacks using the "Threshold" tool to outline only the pixels of the image with $S100\beta^{+}$ staining. Then using the "Measure" tool we calculated the area fraction, the percentage of pixels in the image that have been highlighted using the threshold, and the integrated density, which is the sum of all the pixel intensities (in arbitrary units). Both measures were calculated from at least 5 z-stack of 12 μm of thickness. A minimum of five hippocampal sections were analyzed per animal.

3D-Sholl analysis.

3D Sholl-analysis was performed as previously described (Martín-Suárez et al., 2019). At least 50 individual cells were analyzed after acquiring 20 μm-thick z-stacks, captured with the 63x objective, by a confocal microscope, making sure they contained the whole cell, taken form WT and DS mice brain sections. We used the Sholl open-source plug-in for FIJI (Image J) (Schindelin et al., 2012).This plug-in performs the Sholl technique directly on z-stacks of fluorescence-labeled cells. It is based on an algorithm to retrieve data from pixel-based connectivity (detailed in the user guide of http://fiji.sc/Sholl).

Quantification of apoptosis and microglial phagocytosis.

Apoptosis and microglia phagocytosis were measured as previously described (Abiega et al., 2016). All parameters were measured in the SGZ + GCL of the DG. Apoptosis was assessed by DAPI staining and quantification of the number of pyknotic/ karyorrhectic (condensed/fragmented DNA) nuclei was carried out. Microglial cells were quantified as the number of microglial somas with identifiable nucleus. Phagocytosis was defined as an apoptotic cell fully surrounded (engulfed) by a microglia pouch belonging to a microglial cell (Iba 1^{+}). To estimate the density of apoptotic, phagocytosed and microglial cells, we quantified their numbers inside the granular cell layer volume in each z-stack. Volume was obtained by multiplying the granular cell layer area in the center of the stack with the thickness of the z-stack using ImageJ (Fiji). Three z-stacks were quantified per hippocampal section and 4 sections were quantified per animal. Densities were given in mm. Microglial phagocytic index (Ph index) was estimated using the following formula: Ph index = $\frac{apo^{Ph}}{apo^{tot}}$ where apo^{Ph} is the number of apoptotic cells phagocytosed and apo^{tot} is the total number of apoptotic cells.

Statistical analysis

SigmaPlot (San Jose, CA, USA) was used for statistical analysis. A Student´s t test was performed in all cases to compare data form WT to DS mice for all quantifications except for data from the 3D-Sholl analysis in which a repeated-measures two-way ANOVA with a Bonferroni post-hoc test was employed. When variances were not homogeneous (by Levene´s test) a Mann-Whitney rank sum test was used instead. This case only happened for the data of neuroblasts with basal dendrites. Only $p<0.05$ is reported to be significant. Data are shown as mean ± standard error of the mean (SEM).

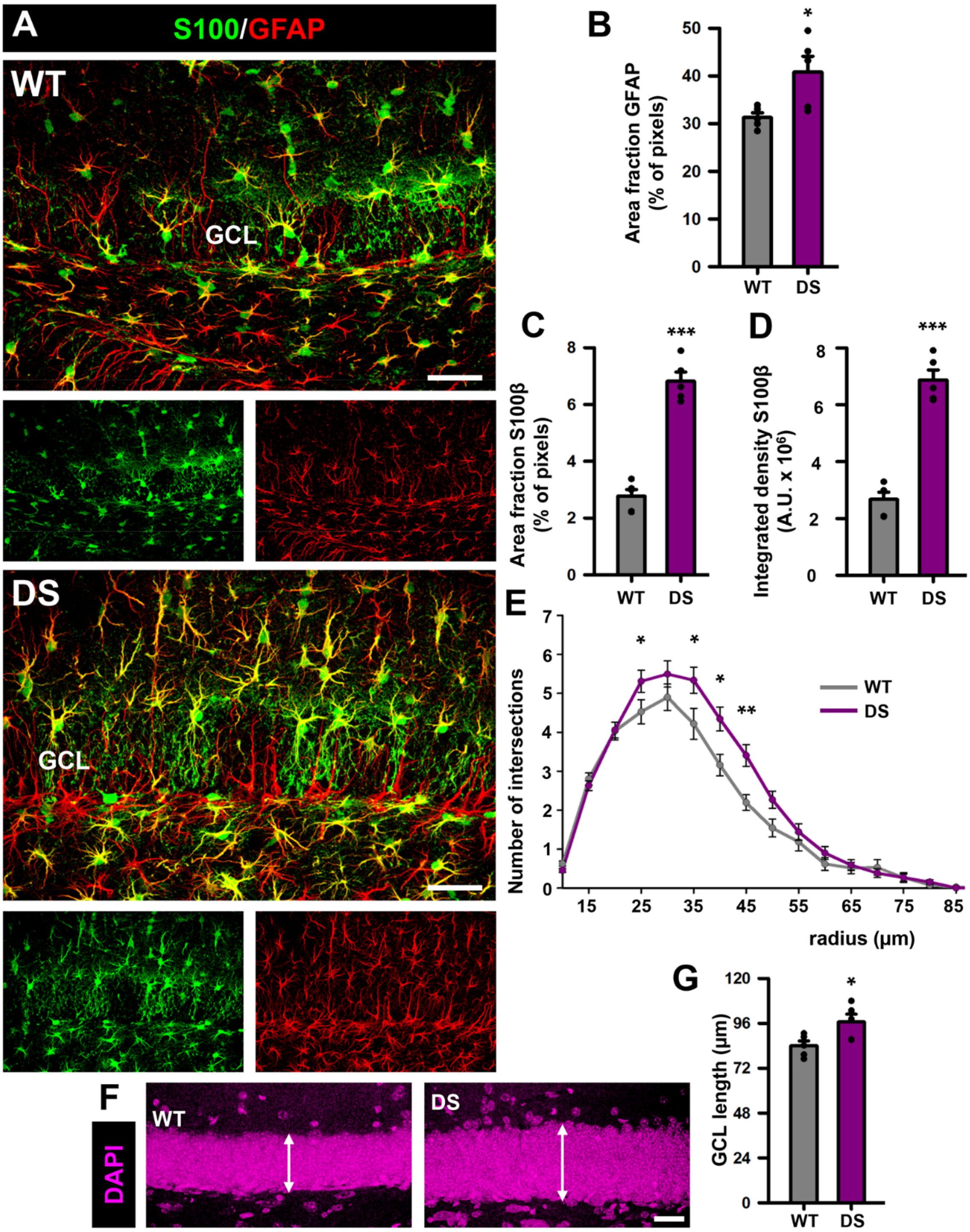

Supplementary Figure 1. Increased gliosis in the neurogenic niche of DS mice. The marker of astrocytes and NSCs GFAP as well as the marker of astrocytes S100β were analyzed in the SGZ and GCL of WT and DS mice **(A)**. The percentage of area occupied by GFAP+-pixels (**B**) was higher in DS mice. The percentage of area occupied by S100β^{+}-pixels **(C)**, as well as the integrated area (pixels multiplied by the intensity of the signal) **(D)** were higher in DS mice. The morphological complexity of S100β^{+}-cells, assessed by 3D-Sholl analysis was also increased in DS mice **(E)**. Using DAPI to stain cell nuclei **(F)** we found that the GCL of DS mice span a larger distance, from the hilus to the molecular layer **(G)**. $*p < 0.05$, $***p < 0.001$ by Student's t-test **(B, C, D and G)**. $*p < 0.05$, $**p < 0.01$, by repeated-measures two-way ANOVA followed by Bonferroni post hoc test in **(E)**. Bars show mean ± SEM. Dots show individual data. Scale bar in **(A)** is 20 µm and 40 µm in **(F)**.

References

Abiega, O., Beccari, S., Diaz-Aparicio, I., Nadjar, A., Layé, S., Leyrolle, Q., et al. (2016). Neuronal Hyperactivity Disturbs ATP Microgradients, Impairs Microglial Motility, and Reduces Phagocytic Receptor Expression Triggering Apoptosis/Microglial Phagocytosis Uncoupling. *PLOS Biology* 14, e1002466. doi:10.1371/journal.pbio.1002466.

Encinas, J. M., and Enikolopov, G. (2008). "Identifying and Quantitating Neural Stem and Progenitor Cells in the Adult Brain," in *Methods in Cell Biology* (Elsevier), 243–272. doi:10.1016/S0091-679X(08)85011-X.

Encinas, J. M., Michurina, T. V., Peunova, N., Park, J.-H., Tordo, J., Peterson, D. A., et al. (2011). Division-coupled astrocytic differentiation and age-related depletion of neural stem cells in the adult hippocampus. *Cell Stem Cell* 8, 566–579. doi:10.1016/j.stem.2011.03.010.

Martín-Suárez, S., Valero, J., Muro-García, T., and Encinas, J. M. (2019). Phenotypical and functional heterogeneity of neural stem cells in the aged hippocampus. *Aging Cell* 18. doi:10.1111/acel.12958.

Ricobaraza, A., Mora-Jimenez, L., Puerta, E., Sanchez-Carpintero, R., Mingorance, A., Artieda, J., et al. (2019). Epilepsy and neuropsychiatric comorbidities in mice carrying a recurrent Dravet syndrome SCN1A missense mutation. *Scientific Reports* 9. doi:10.1038/s41598-019-50627-w.

Schindelin, J., Arganda-Carreras, I., Frise, E., Kaynig, V., Longair, M., Pietzsch, T., et al. (2012). Fiji: an open-source platform for biological-image analysis. *Nature Methods* 9, 676–682. doi:10.1038/nmeth.2019.
